# Mechanism-Driven Design of Metal–Phenolic Networks: From Precision Assembly to Multidimensional Synergistic Therapy for Brain Diseases

**DOI:** 10.3390/pharmaceutics18091192

**Published:** 2026-09-21

**Authors:** Mengyue Ni, Bo Hu, Di Zhu, Boya Li, Yijiang Jia, Yu Lu, Yuji Wang

**Affiliations:** 1 Department of Medicinal Chemistry, College of Pharmaceutical Sciences of Capital Medical University, Beijing 100069, China; 2 Beijing Key Laboratory of Intelligent Clinical Data and AI-Driven Innovative Drug Development, Beijing Laboratory of Biomedical Materials, Laboratory for Clinical Medicine, Capital Medical University, Beijing 100069, China; 3 Department of Pharmacy, Xuanwu Hospital of Capital Medical University, Beijing 100053, China

**Keywords:** metal phenolic networks, Alzheimer’s disease, glioma, stroke, NETs

## Abstract

**Background**: Brain disorders, such as glioblastoma, Alzheimer’s disease and ischemic stroke, represent formidable challenges due to the highly restrictive blood–brain barrier (BBB) and the complex pathophysiological microenvironment, which severely compromise drug delivery and therapeutic outcomes. Metal–phenolic networks (MPNs), as a class of novel hybrid materials, hold great promise for overcoming brain delivery bottlenecks and enabling precise synergistic therapy by leveraging their unique physicochemical properties. However, a comprehensive synthesis of MPN designs tailored specifically to the distinct pathological mechanisms underlying brain disorders is currently lacking. **Methods**: In this review, we systematically outline the foundational building blocks of MPNs and their diverse nanostructure engineering strategies. Building on this, we closely align MPN customization with the four core pathological pillars of brain diseases, thoroughly summarizing advanced design strategies and synergistic therapeutic mechanisms. Additionally, we prospectively discuss emerging innovative approaches, including the targeting of neutrophil extracellular traps (NETs) to modulate the immunosuppressive tumor microenvironment. **Outlook**: Finally, we critically examine the pivotal obstacles hindering the clinical translation of MPNs, particularly concerning uncertainties in long-term biosafety and in vivo metabolism, metal-specific neurotoxicity and coordination-stability-governed metal release kinetics, challenges in quality control during scalable production, and the unpredictable interference of the protein corona. **Conclusions**: In this review, we provide a robust theoretical foundation for the rational design of mechanism-driven MPNs, while offering forward-looking perspectives to accelerate their clinical translation from bench to bedside for the precise management of brain diseases.

## 1. Introduction

Brain diseases, including Alzheimer’s disease (AD), stroke, and glioma (GBM), pose significant global public health challenges due to their high mortality, high disability rates, and the heavy socioeconomic burdens that they impose [1]. However, the high selectivity of the blood–brain barrier (BBB) and the unique pathological microenvironment of brain diseases severely impede the entry of therapeutic agents into the lesions, further compromising their pharmacological efficacy [2].

In recent years, metal–phenolic networks (MPNs) have garnered increasing attention due to their self-assembly characteristics, favorable biocompatibility, and pH-responsive properties [3]. However, current research in this field remains fragmented. Huang et al. [4] discussed the latest advances and design principles of MPNs in cancer therapy and explored their potential as multifunctional therapeutic platforms, while also outlining the key challenges in translating MPNs into clinical practice. Wang et al. [5] discussed the mechanisms of MPNs in treating chronic wounds and their applications in novel chronic wound therapies. Liang et al. [6] summarized the applications of MPNs in repairing and overcoming various biological barriers, including the blood–brain barrier, pulmonary mucosal barrier, gastrointestinal barrier, skin barrier, and tumor barrier. However, to date, no review has specifically addressed the application of MPNs in brain diseases. Consequently, there remains a lack of comprehensive and in-depth summaries regarding how MPNs can be designed to cross the BBB, how their structural tunability can be leveraged to actively adapt to the brain disease microenvironment, and the systematic translational pathways from fundamental research to clinical practice.

In light of this, we aim to systematically summarize the latest research progress regarding metal–phenolic networks (MPNs) in the treatment of brain diseases. We first elaborate on the fundamental building blocks, assembly mechanisms, and unique physicochemical properties of MPNs. Subsequently, we highlight and synthesize the cutting-edge applications of MPNs in targeted glioma therapy, neurodegenerative disease intervention, and stroke neuroprotection, while providing an in-depth analysis of their underlying therapeutic mechanisms. Finally, we discuss the current challenges facing this field and the prospects for future clinical translation.

In this review, we not only provide a robust theoretical foundation for the development of MPN-based precision diagnostic and therapeutic strategies for brain diseases but, more importantly, offer forward-looking guidance for researchers in pharmacology and neuroscience by critically dissecting the core bottlenecks currently hindering clinical translation. Consequently, this review holds profound scientific value and practical significance for advancing the translation of MPNs from the laboratory bench to clinical practice.

## 2. Foundations and Physicochemical Properties of MPNs

Metal–phenolic networks (MPNs) are amorphous coordination networks self-assembled from metal ions (nodes) and phenolic ligands (bridging molecules) [7]. Their physicochemical properties and biological functions are highly dependent on the rational selection of building blocks. A key advantage of MPNs is that both the metal ions and polyphenols constituting the network are therapeutically active components [8]. When integrated with various functional components, they yield composite materials with significant potential in chemistry, biology, and materials science. Owing to their remarkable physicochemical properties, MPNs have been extensively studied for their biomedical applications, including bioimaging, drug delivery, and surface coatings [7]. Typically, MPNs are synthesized by dissolving polyphenol salts in alcohol or aqueous solutions, followed by the addition of metal salts. This reaction can be carried out under varying stirring and/or heating conditions. A base is generally employed to deprotonate the hydroxyl groups and promote metal coordination. The resulting products precipitate from the solution and are subsequently collected via filtration or centrifugation, followed by air-drying [9].

**Figure 1:**
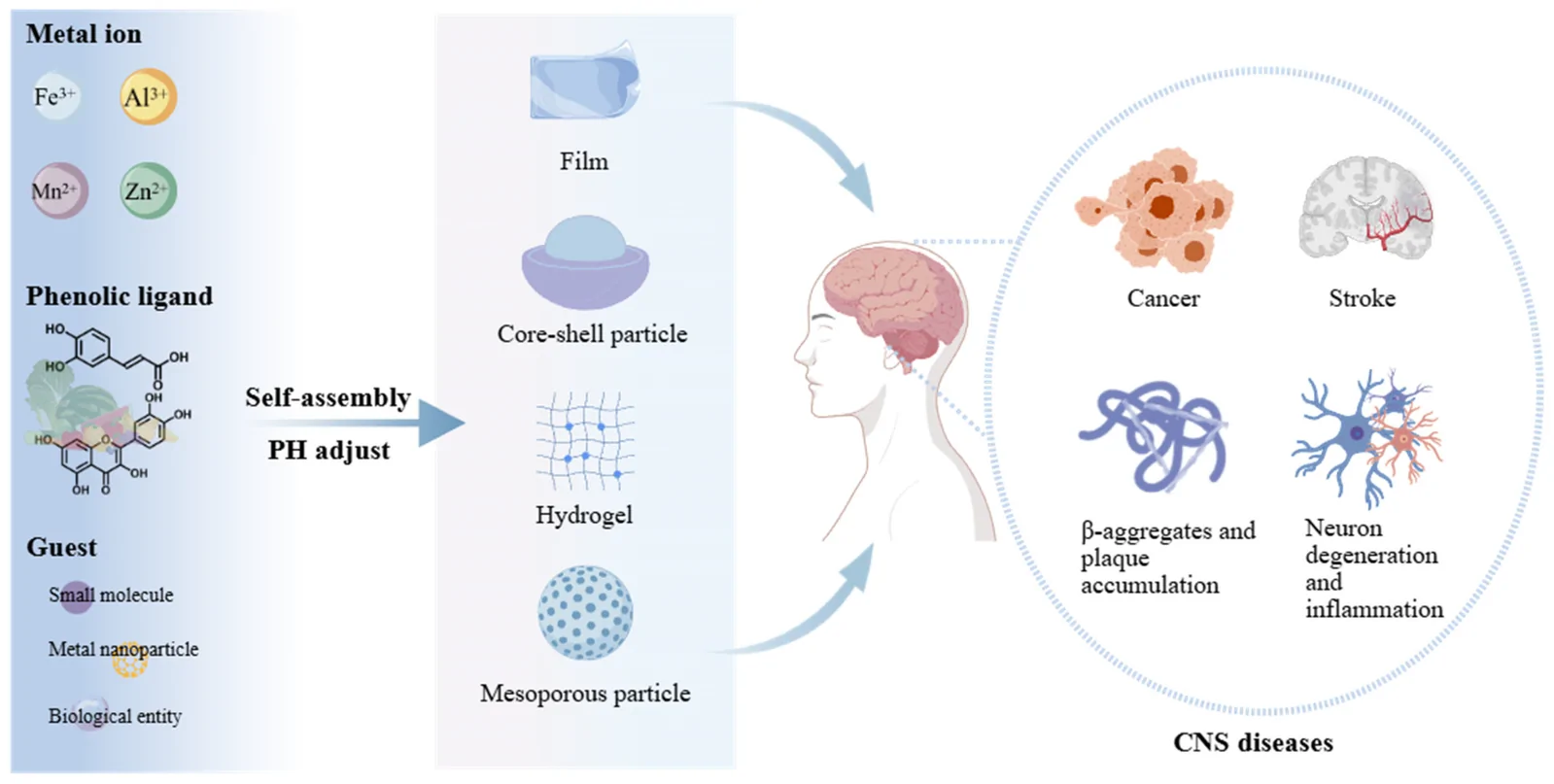
A schematic illustration of the building blocks, morphologies, and applications in brain diseases.

Through nanostructure design, self-assembled metal–phenolic networks can be tailored for diverse applications, as illustrated in Figure 1.

### 2.1. Function-Oriented Selection of Metal Ion Nodes

Metal ions are the primary components of MPNs (Table 1). Main-group metal ions (e.g., aluminum), transition metal ions (e.g., iron, copper, cadmium), and rare-earth ions (e.g., Ce, Eu, Sm, and Gd) have been widely employed in preparing MPNs [10]. The type of metal ion and its stoichiometric ratio dictate MPN properties such as film thickness, permeability, and disassembly characteristics. Furthermore, the selection of metal ions determines the specific functions of MPNs [11,12].

### 2.2. Critical Considerations for Metal Selection in CNS Applications

However, the benefits of these metals must be weighed against their metal-specific neurotoxicity, which is dependent on dose, oxidation state, and release kinetics and is particularly consequential in the central nervous system (CNS) [27]. Redox-active metal ions such as Fe and Cu can catalyze Fenton and Fenton-like reactions; once released in an uncontrolled manner, they acquire pro-oxidant activity and exacerbate oxidative damage. Mn similarly exhibits a dose–effect duality: although Mn^2+^ mimics antioxidant enzymes and activates cGAS–STING signaling at pharmacological doses, chronic Mn exposure is a well-established cause of manganism because circulating Mn^2+^ enters the brain via transferrin receptor- and divalent metal transporter 1-mediated routes and accumulates preferentially in the globus pallidus [28]. Among rare-earth metals, the deposition of Gd in the dentate nucleus and globus pallidus observed after repeated administration of gadolinium-based contrast agents constitutes a cautionary precedent for chronic dosing regimens [29]. Even essential metals display dose-dependent effects: Zn^2+^ is neuroprotective and competitively blocks Cu/Fe-induced Aβ aggregation at physiological concentrations, whereas zinc overload promotes Aβ deposition and synaptic dysfunction [30]. Taken together, these considerations argue for optimizing metal dose, oxidation state, and release kinetics for every CNS-targeted MPN, rather than selecting metal ions solely for maximal catalytic or imaging performance.

On the basis of this toxicity–activity balance, we propose that, for CNS applications, the metal ions in MPNs should be selected according to disease-specific characteristics. First, when cytotoxic pro-oxidation is itself the therapeutic objective, redox-active ions (Fe^3+^/Fe^2+^, Cu^2+^/Cu^+^, Mn^2+^) are justified for glioblastoma therapy [31,32]. Second, in Alzheimer’s disease, where excess Cu, Fe, and Zn are pathological drivers rather than therapeutic agents, the MPN should be designed such that its polyphenolic ligands act as metal chelators to sequester labile metal ions and block their binding to Aβ [33]. Third, when the MPN serves mainly as a carrier or coating, redox-inert ions (Zn^2+^, Mg^2+^, Sr^2+^, Ca^2+^) are preferred, providing biological functions without participating in redox cycling. Collectively, on the premise of ensuring metal activity, the associated toxicity can be controlled to a certain extent.

### 2.3. Endogenous Pharmacological Activities and Surface Modification of Phenolic Ligands

**Figure 2:**
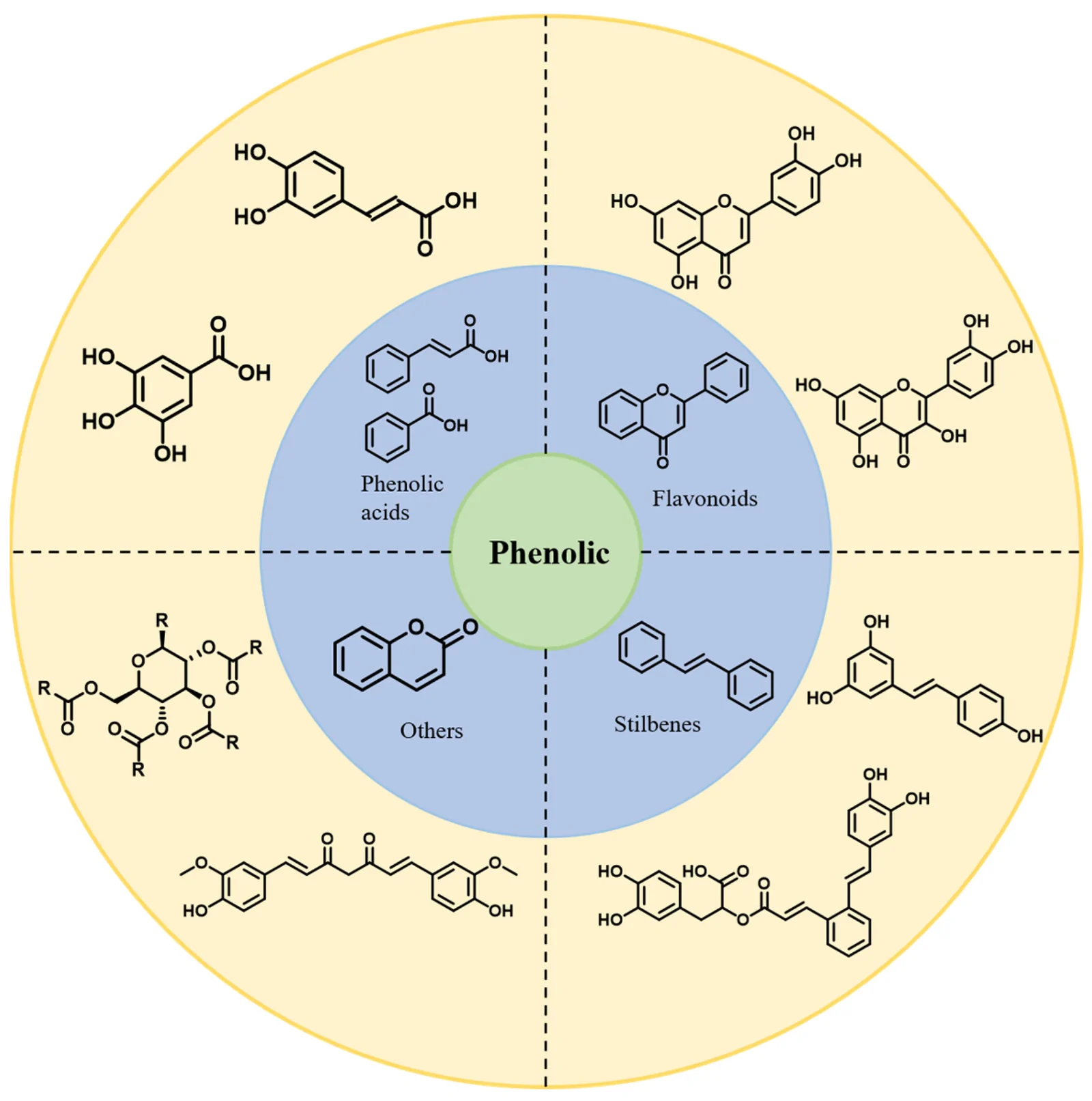
The chemical structures of polyphenols applied in metal–phenolic networks (MPNs).

Polyphenols, as integral components of MPNs, endow these networks with diverse pharmacological activities, as illustrated in Figure 2. For instance, compounds such as curcumin, quercetin, resveratrol, phytol, thymoquinone, ginsenosides, and huperzine A have been demonstrated to possess antioxidant, anti-inflammatory, Aβ aggregation-inhibitory, and cholinesterase-inhibitory properties [34].

Pharmacological studies indicate that some polyphenols and flavonoids—the molecular building blocks of MPNs—can cross the blood–brain barrier (BBB), a property tentatively attributed to their lipophilicity and low molecular weight. However, it should be noted that this permeability should not be equated with the ability of intact MPN nanoparticles to traverse the BBB, which requires separate experimental verification [35,36].

Furthermore, polyphenols offer advantageous conditions for brain-targeting applications of MPNs [37,38,39]. Through structural modifications of polyphenols, such as surface PEGylation, the conjugation of targeting ligands, or further modification with brain-targeting natural products (e.g., borneol [40,41], menthol [42,43,44], muskone [45], and asarone [46,47]), active brain targeting and prolonged circulation time can be achieved. Additionally, introducing boronate ester groups into the structure enables the construction of highly ROS-sensitive release switches [48].

The functions of phenolic ligands can also be tailored via chemical modification. For example, hyaluronic acid (HA)-based phenolic ligands can be used for cell targeting [49,50]. By altering the ratio of dopamine-modified polymeric phenolic ligands, MPN composites with tunable cell-targeting effects—such as those incorporating HA or polyethylene glycol (PEG)—can be prepared. Furthermore, dopamine can be employed to endow various molecules with catechol groups, thereby enhancing their underwater adhesion properties [51,52].

However, the clinical application of these natural compounds has long been constrained by pharmacokinetic drawbacks, including low solubility, poor bioavailability, and rapid in vivo metabolism [53]. Formulating these compounds as MPN nanoformulations can effectively enhance brain delivery in selected preclinical models. Extensive preclinical studies have demonstrated that nanoformulations loaded with such natural compounds significantly outperform free drugs in reducing Aβ deposition, inhibiting tau protein phosphorylation, and alleviating oxidative stress and neuroinflammation in models such as Alzheimer’s disease [54,55]. Therefore, MPNs not only inherit the therapeutic activities of polyphenols but also resolve their druggability challenges through nano-engineering, achieving a combined effect [8].

### 2.4. Nanostructural Design of MPNs

Although the synthesis process is straightforward, careful nanostructural design is ultimately required to achieve optimal drug loading and release profiles or to adapt the platforms to different types of disease.

#### 2.4.1. Film Coatings

Film coatings [56] are nanoscale functional shells formed on the surfaces of other nanoparticles or materials via coordination between metal ions and phenolic ligands. This coating strategy aims to functionally modify the encapsulated nanomaterials and can be applied to virtually any nanoparticle or substrate.

**Figure 3:**
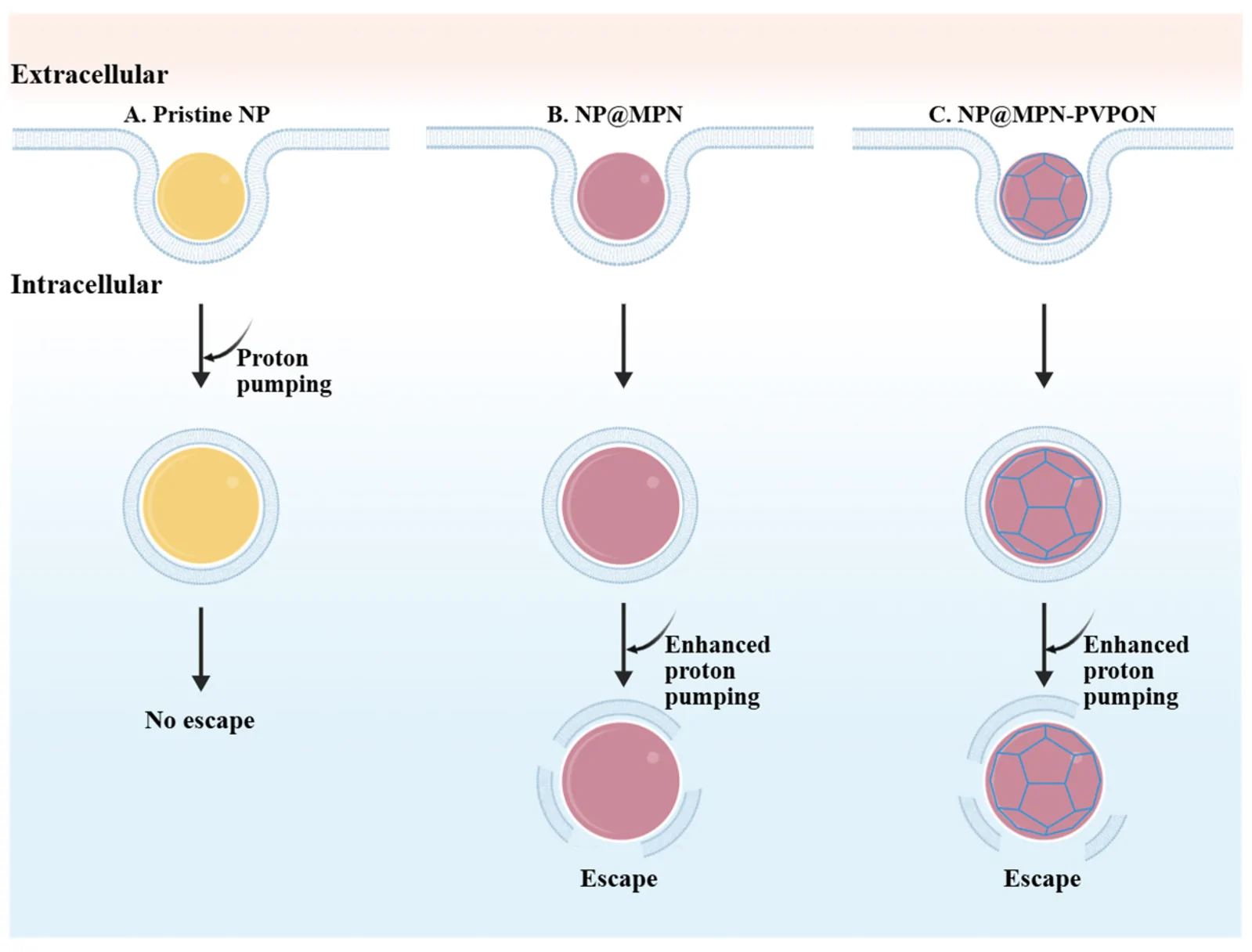
MPN facilitates endosomal escape via the “Proton-Sponge Effect”: (**A**) Pristine nanoparticles (NPs) struggle to escape from endo-/lysosomes. (**B**) MPN-coated NPs (NP@MPN) escape from endo-/lysosomes. (**C**) Postfunctionalizing NP@MPN with a poly(*N*-vinylpyrrolidone) (PVPON) layer does not impact the ability to escape the endosome, indicating that escape is not mediated by membrane disruption.

Chen et al. [57] coated nanoparticles with a metal–phenolic network (MPN) layer, leveraging its pH-responsive disassembly within the acidic endosomal environment to trigger endosomal escape and subsequently release the encapsulated drugs into the cytosol (Figure 3). The MPN coating is formed via the coordination-driven self-assembly of metal ions (e.g., Fe^3+^, Al^3+^) and phenolic ligands (e.g., tannic acid, TA). The coordination bonds remain stable at physiological pH (7.4) but undergo protonation-induced cleavage in the acidic endosomal environment (pH 5.0–6.5), leading to disassembly of the coating. Upon disintegration, the uncoordinated phenolic hydroxyl groups extensively bind protons in the acidic environment. The ensuing osmotic imbalance—by analogy with the “proton-sponge hypothesis”—is thought to be accompanied by a massive influx of Cl^−^ and water molecules, ultimately resulting in osmotic swelling, membrane rupture, and increased membrane permeability. It should be noted that the proton-sponge concept itself remains a debated hypothesis in the nanocarrier field [58], and specifically for MPN coatings, direct measurements of endosomal pH, membrane integrity, and quantitative cytosolic delivery have not yet been reported; current evidence is largely indirect. In the study, the authors demonstrated the universality of the MPN coating strategy, forming uniform layers on various nanoparticles, including polymeric nanoparticles, mesoporous silica nanoparticles, gold nanoparticles, and protein nanoparticles.

Although the study was not specifically focused on brain diseases, it holds significant reference value for drug delivery to the brain. Even if nanoparticles successfully traverse the BBB, they must still enter the cytosol of neurons or glial cells to exert their effects. The endosomal escape function provided by the MPN coating can be used to address this delivery challenge. Furthermore, the abnormal intracellular aggregation of tau protein in Alzheimer’s disease (AD) and the cytosolic/nuclear targets of chemotherapeutic agents in glioma could both benefit from the endosomal escape capability of MPN coatings.

Yang et al. [59] achieved the super-assembly of mesoporous silica nanoparticles (MSNs) with a metal–phenolic network (MPN), loading anticancer drugs into the MSN mesopores. The outer MPN coating endowed the system with dual responsiveness to photothermal stimuli and variations in pH. Under near-infrared (NIR) irradiation, the MSN@MPN nanoplatform exhibited excellent photothermal effects and superior pH-triggered drug release profiles, demonstrating remarkable biocompatibility while effectively killing tumor cells.

#### 2.4.2. Core–Shell Structures

Core–shell structures [60] refer to composite nanoparticle systems composed of a central core and an outer shell layer. This represents an integrated structural design where the core and shell work synergistically to form a complete drug nanosystem.

**Figure 4:**
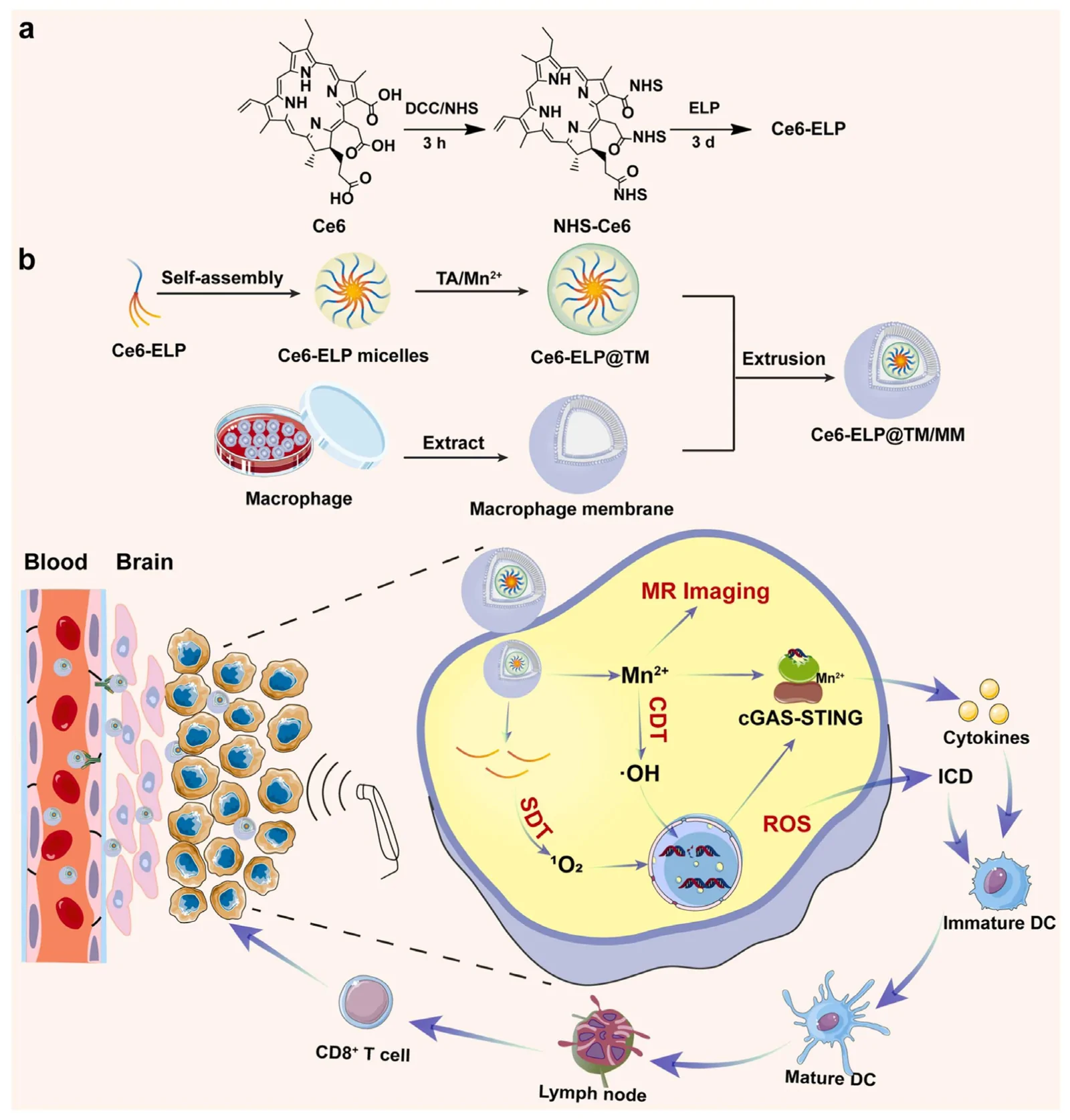
A schematic illustration of the preparation of Ce6-ELP@TM/macrophage membrane (MM) for magnetic resonance (MR)/fluorescence imaging and combination therapy of orthotopic glioma. (**a**) Synthetic pathway of Ce6-ELP. (**b**) A biomimetic MPN-coated ELP micellar platform as an ICD inducer for orthotopic glioma SDT/CDT/immune therapy.

Wang et al. [61] designed and constructed a macrophage membrane-biomimetic metal–polyphenol network-coated elastin-like polypeptide micelle for in situ sonodynamic/chemodynamic/immunotherapy of glioma (Figure 4). The research team linked the sonosensitizer chlorin e6 (Ce6) to an ELP (molecular weight: 33.7 kDa; amino acid sequence: MW[VPGVG-VPGMG-(VPGVG)_2_]_20_) via covalent bonds, which self-assembled into nanomicelles in aqueous solution. These were subsequently coated with an MPN composed of tannic acid and Mn^2+^ to form Ce6-ELP@TM. Finally, the Ce6-ELP@TM was camouflaged with macrophage membranes (MMs) to obtain the final nanodrug, Ce6-ELP@TM/MM. Within this platform, the MPN demonstrated potential for tumor chemodynamic therapy (CDT) and magnetic resonance (MR) imaging, exhibiting excellent biocompatibility, pH sensitivity, and CDT performance. Furthermore, Mn^2+^ enhanced the sensitivity of cGAS to DNA and the affinity of cGAMP for STING, synergizing with CDT/SDT to effectively activate antitumor immune responses.

### 2.5. Other Potential Nanomaterial-Based Strategies

#### 2.5.1. Hydrogels

Currently, numerous studies have designed MPN-based hydrogels [62], primarily leveraging their anti-inflammatory and antioxidant properties in synergy with the hydrogel matrix to promote wound or tissue repair.

For instance, MPN-integrated hydrogels have been developed for diabetic wound healing [63], periodontal repair [64], and bone defect regeneration [65]. These applications exploit the MPN’s ability to scavenge ROS, suppress inflammatory cytokines, and promote tissue integration.

Although direct applications of MPN-based hydrogels for brain diseases have not yet been reported, these studies offer design concepts that could potentially be adapted for CNS applications. For example, the pH-responsive and ROS-scavenging properties demonstrated in wound-healing MPN hydrogels could be translated to the acidic, oxidative microenvironment of ischemic stroke or traumatic brain injury, where injectable hydrogels might provide sustained local delivery of neuroprotective agents. However, such CNS applications remain speculative and require dedicated validation in appropriate brain disease models.

#### 2.5.2. Capsules

MPN capsules have also been developed, primarily leveraging their drug-loading capabilities for intratumoral drug release or pulmonary delivery.

For example, nebulized MPN capsules have been used for controlled pulmonary deposition to achieve targeted deposition in different regions of the respiratory tract [66]; microcapsules with dual functions of drug loading and immunomodulation have been constructed to inhibit the growth of both primary and distant tumors [67].

Although no studies have reported the application of MPN capsules in brain diseases, their characteristics—including immunomodulatory activity, drug-loading capacity, controlled release, and nanoscale size—endow them with potential for application in brain diseases.

## 3. Mechanism-Driven MPN Design Strategies for Brain Diseases

**Figure 5:**
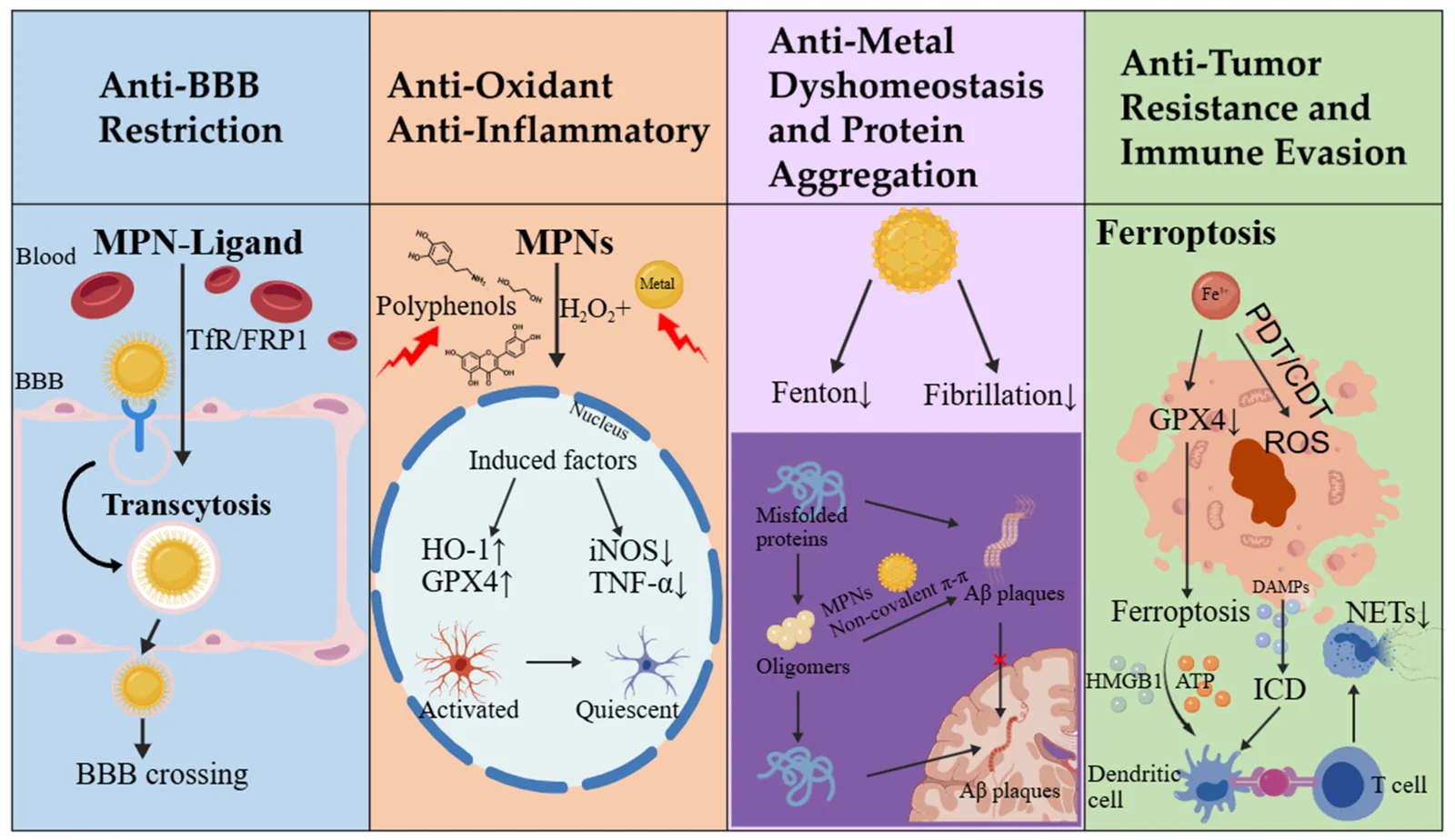
The four core mechanisms of metal–phenolic networks (MPNs).

Precisely matching MPN design with the pathogenesis of brain diseases is central to achieving efficient synergistic therapy. Although brain diseases are diverse, their pathological processes often involve several common, interconnected destructive pathways (Figure 5): the BBB’s hindrance of drug delivery, the vicious cycle of oxidative stress and neuroinflammation, the exacerbation of abnormal protein aggregation due to metal ion homeostasis imbalance, and tumor drug resistance and immune evasion. In the following sections, we will elaborate on customized MPN design strategies based on these four core mechanisms and their applications in specific diseases, including neurodegenerative disorders, brain tumors, and stroke. The strategies discussed below differ substantially in the strength of their supporting evidence, ranging from direct demonstration of intact-particle BBB crossing to increased brain accumulation and to mechanisms inferred primarily from design rationale.

### 3.1. BBB Penetration Strategies and MPN Design

The greatest challenge in treating diseases of the central nervous system (CNS) lies in the low drug transport efficiency caused by the blood–brain barrier (BBB) [68].

The blood–brain barrier (BBB) is a semipermeable barrier that envelops the microvessels of the central nervous system (CNS), regulating the entry and efflux of molecules between the vascular compartment and the brain [69]. While the BBB effectively prevents most blood-borne substances from entering the brain, this protective function also excludes over 98% of small-molecule drugs and virtually all macromolecular therapeutics from penetrating the brain, thereby severely limiting the pharmacological efficacy of treatments for brain diseases [70,71].

**Figure 6:**
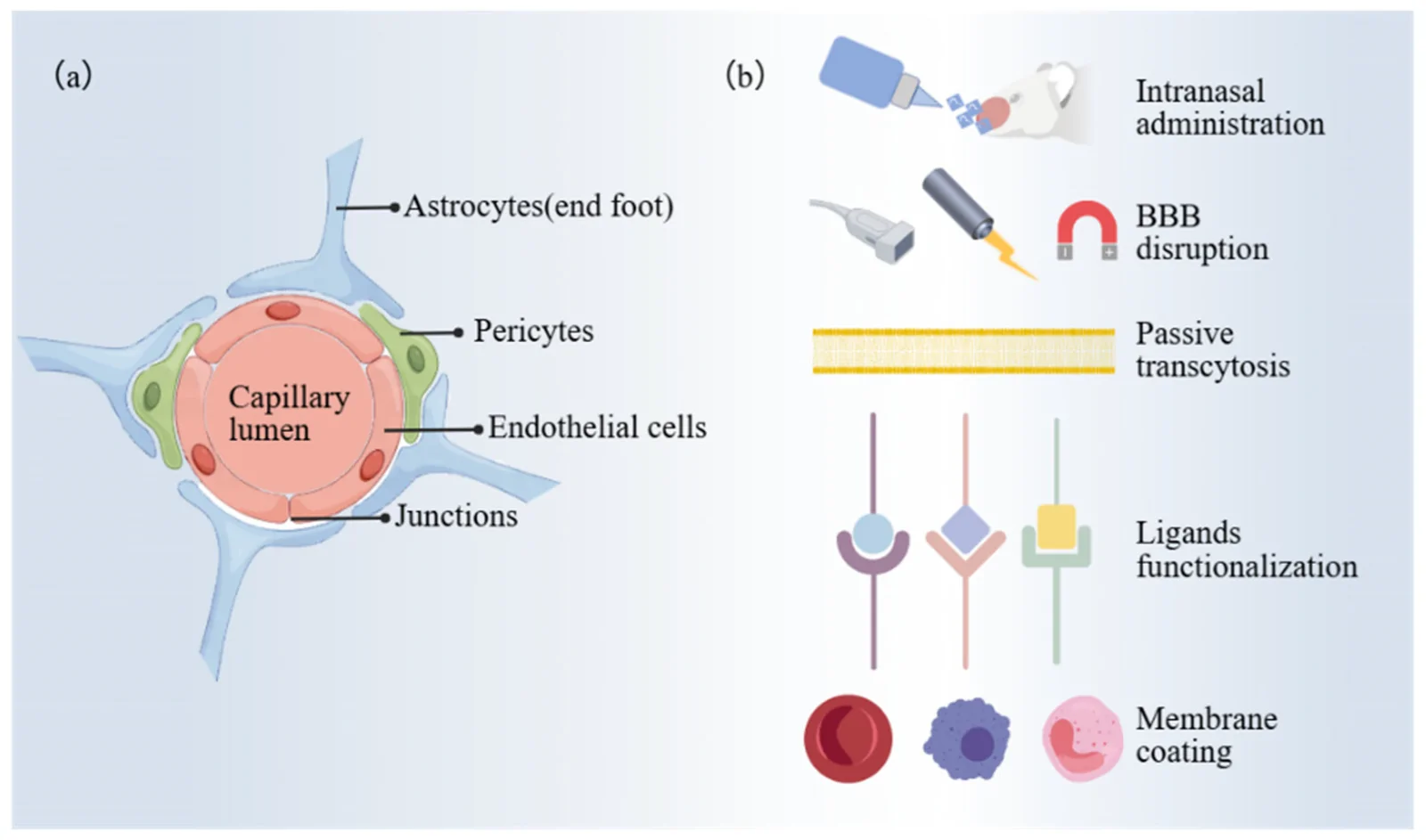
Strategies and materials for modulating the blood–brain barrier (BBB) and brain-targeted drug delivery: (**a**) A schematic diagram depicting the structural composition of the BBB. (**b**) Engineering materials employed for brain-targeted drug delivery.

Several BBB penetration strategies are currently available(Table 2). Among them, certain strategies can be effectively integrated with MPNs to achieve a synergistic effect, enabling both the traversal of the BBB and brain targeting (Figure 6). (1) Ligand–receptor-mediated transcytosis [72,73,74]: By modifying the MPN surface with specific targeting peptides, transcytosis can be achieved through highly expressed receptors on the BBB, such as the transferrin receptor. (2) Biomimetic membrane coating [75,76]: Coating MPNs with natural biological membranes has been reported to enhance BBB penetration and lesion homing in glioma models; however, whether this reflects active transcytosis or passive retention at the disrupted tumor vasculature has not been definitively resolved [18].

As mentioned above, polyphenolic ligands possess blood–brain barrier (BBB) permeability at the molecular level, but this does not imply that intact MPNs can cross the BBB. Ultrasmall size can improve renal clearance and tumor penetration and may permit passive leakage across compromised barriers, but size reduction alone does not confer BBB-crossing ability in the healthy brain, where transport is governed mainly by receptor-mediated transcytosis and active efflux. The EPR [77,78] effect relies on vascular leakiness and is well documented for peripheral solid tumors; within the CNS it can operate only where the BBB is disrupted—e.g., the leaky core of glioblastoma, the acute phase of ischemic stroke, or advanced neuroinflammatory lesions—and its magnitude and reproducibility in brain tumors remain debated. Therefore, EPR-based brain targeting should not be treated as a universal mechanism. Finally, BBB permeability itself differs substantially among disease models: the healthy brain retains an intact barrier; AD is characterized by focal barrier dysfunction and transporter alterations; ischemic stroke shows a time-dependent acute breakdown; and glioblastoma presents heterogeneous disruption—each MPN formulation must be evaluated against the barrier status of its specific target disease [79].

### 3.2. Design Strategies Targeting Oxidative Stress and Neuroinflammation

#### 3.2.1. The Vicious Cycle of Oxidative Stress and Inflammation in Stroke and Neurodegenerative Diseases

Oxidative stress is recognized as the primary pathogenic mechanism underlying cerebral ischemia–reperfusion injury (CIRI) [80]. During the acute phase of ischemic stroke, the restoration of blood flow triggers a massive burst of reactive oxygen species (ROS), thereby inducing oxidative stress, neuroinflammation, and neuronal death. This cascade ultimately establishes a self-perpetuating vicious cycle characterized by “ischemia, reperfusion, oxidative damage, inflammation, and further injury” [81].

Similarly, in Alzheimer’s disease (AD), oxidative stress serves as a critical factor that exacerbates Aβ deposition and tau hyperphosphorylation. The aberrant aggregation of Aβ activates microglia, promoting the excessive production of reactive oxygen species (ROS) and pro-inflammatory cytokines. Conversely, the oxidative stress microenvironment further accelerates Aβ generation and aggregation [82,83]. Together with neuroinflammation, this constitutes a reciprocal vicious cycle, ultimately leading to synaptic damage, neuronal loss, and cognitive decline.

Therefore, the timely clearance of ROS and the blockade of inflammatory cascades are pivotal for improving prognosis in stroke and AD, as well as for promoting neural repair.

#### 3.2.2. Dual Microenvironment-Responsive Strategies with Antioxidant Enzyme-Mimicking Activities

In response to the aforementioned vicious cycle of oxidative stress, design strategies for MPNs primarily focus on the following aspects: (1) ROS scavenging via valence variation in metal ions [84]—by exploiting the redox transitions of metal ions such as Mn^2+^ and Fe^2+^ between different oxidation states, MPNs can mimic the activities of superoxide dismutase (SOD) and catalase (CAT), thereby efficiently eliminating superoxide anions and H_2_O_2_. (2) Synergistic antioxidant and anti-inflammatory effects of polyphenolic ligands—polyphenols not only directly scavenge free radicals but also enhance endogenous antioxidant defenses via the Nrf2 pathway while suppressing the NF-κB pathway [85]. This facilitates the polarization of microglia from the pro-inflammatory M1 phenotype to the anti-inflammatory, pro-repair M2 phenotype. (3) pH-responsive dissociation for targeted release—leveraging the acidic microenvironment resulting from anaerobic glycolysis in stroke or ischemic regions, pH-responsive MPNs [86] can be designed to undergo controlled dissociation. This enables the precise release of therapeutics at the lesion site while minimizing systemic side effects.

For instance, Chen et al. [21] utilized the phenolic structure of rutin to coordinate with Mn^2+^ and Sr^2+^ in an alkaline environment, achieving self-assembly into Sr_2_/Mn_2_@Rh bimetallic phenolic network nanoparticles for the treatment of stroke. In this nanoparticle design, Mn^2+^ mimics antioxidant enzyme activities, efficiently scavenging reactive oxygen species (ROS) through its valence-switching properties across oxidation states such as Mn^2+^, Mn^3+^, and Mn^4+^. Meanwhile, Sr^2+^ regulates endothelial cell proliferation, thereby promoting angiogenesis and improving vascular function. The antioxidant activity of rutin, acting synergistically with the metal ions, effectively eliminates the massive burst of ROS triggered by ischemia–reperfusion injury.

This metal–phenolic network remains stable under neutral and alkaline conditions but undergoes dissociation specifically within the acidic microenvironment of ischemic brain tissue, thereby enabling the targeted release of rutin, Mn^2+^, and Sr^2+^. Mechanistically, this platform effectively mitigates neuroinflammation by promoting the phenotypic transition of microglia from the pro-inflammatory M1 phenotype to the anti-inflammatory, pro-repair M2 phenotype, which is characterized by significant downregulation of iNOS expression and upregulation of CD206 expression. Additionally, Mn^2+^ and Sr^2+^, acting as weakly alkaline ions, neutralize the accumulated lactic acid in ischemic tissues, thereby alleviating intracellular acidosis and simultaneously promoting vascular normalization.

Hou et al. [87] developed a nanocomposite (GQDs@MPN) in which graphene quantum dots (GQDs) were the core, and they were coated with a metal–phenolic network formed via the coordination of epigallocatechin gallate (EGCG) and Co^2+^. The GQDs, serving as a nanocarrier, exhibited favorable biocompatibility and ultrasmall size, which was proposed to facilitate blood–brain barrier (BBB) transport. Although experimental results have shown increased brain accumulation in vivo, direct evidence of intact-particle BBB crossing remains limited. The GQDs@MPN system leveraged the Co-EGCG complex to scavenge ROS, upregulate the Nrf2 pathway, and suppress the NF-κB pathway, thereby activating downstream antioxidant enzyme systems. This approach successfully alleviated neuroinflammation and improved cognitive function in the APP/PS1 mouse model of Alzheimer’s disease.

Furthermore, Liu et al. [88] advanced this strategy by designing the TQCN nanocomposite, which achieved mitochondrial targeting via triphenylphosphonium (TPP) modification. This platform utilized the coordination network formed between quercetin and iron ions to chelate excess iron, thereby inhibiting the Fenton reaction. Concurrently, it activated the Nrf2 pathway to enhance the activity of antioxidant enzymes such as GPX4. Through this dual mechanism of exogenous iron chelation and endogenous antioxidant defense, the TQCN nanocomposite effectively inhibited the ferroptosis-driven pathological progression of AD.

### 3.3. Interventions Against Metal Ion Dyshomeostasis and Pathological Protein Aggregation

#### 3.3.1. Metal Overload and Aβ/Tau Aggregation in Alzheimer’s Disease: Pathogenic Interplay

Alzheimer’s disease (AD) is a complex, multifactorial disorder that cannot be effectively treated by modulating a single biological target [89]. Its major pathological hallmarks include amyloid-β (Aβ) plaque deposition, tau neurofibrillary tangles, and aberrant overload of transition metals (e.g., Cu^2+^, Fe^3+^) within the brain. Studies have revealed significantly increased levels of Cu^2+^, Fe^3+^, and Zn^2+^ in the cerebral cortex and hippocampus of AD patients [90]. These excessive metal ions not only exacerbate oxidative stress via the Fenton reaction but also directly promote Aβ aggregation. Therefore, maintaining metal ion homeostasis while simultaneously regulating Aβ aggregation and oxidative stress represents a more effective therapeutic direction [91].

#### 3.3.2. Natural Chelation and Aggregation-Inhibitory Effects of Polyphenol-Metal Coordination Networks

Addressing the aforementioned multiple pathological mechanisms, MPNs offer unique therapeutic advantages, with their design strategies primarily encompassing the following three aspects: (1) Inhibition of Aβ aggregation by polyphenols—The phenolic ligands within MPNs can act as antagonists of Aβ aggregation. Plant-derived polyphenols (e.g., EGCG) bind to Aβ through hydrophobic interactions and hydrogen bonding, thereby inhibiting its aggregation [92]. Specifically, EGCG, which contains multiple phenolic hydroxyl groups and aromatic rings, can suppress Aβ fibrillization via non-covalent interactions. These actions enable polyphenols to adsorb and stabilize Aβ monomers or oligomers, preventing their conformational transition into β-sheet-rich structures [93]. (2) Synergistic anti-inflammatory and antioxidant effects—Polyphenols inherently scavenge free radicals and suppress the NF-κB pathway to reduce neuroinflammation, thereby creating a favorable microenvironment for subsequent Aβ clearance [94]. (3) Metal coordination-enhanced polyphenol functions—Metal ion complexation not only protects polyphenols from auto-oxidation, thereby improving their stability and bioavailability, but also significantly augments their antioxidant and protein aggregation-inhibitory capabilities. Studies have demonstrated that metal–phenolic networks exhibit superior radical-scavenging efficiency compared to free polyphenols. For instance, rare-earth-metal complexes increased the superoxide anion radical (O_2_•^−^) scavenging activity of quercetin by an average of twofold [95]; similarly, the complexation of metals with chrysin increased ABTS^+^ radical scavenging activity from 0.9 mM to 3.96 mM [96]. This synergistic effect establishes MPNs as a more potent therapeutic intervention platform for AD than free polyphenols alone.

For instance, Zhu et al. [97] employed 9-fluorenylmethoxycarbonyl-tryptophan (Fmoc-Trp), quercetin (Que), and ferric ions (Fe^3+^) as building blocks to fabricate Fmoc-Trp-Fe^3+^-Que nanoparticles (FTFQ NPs) via coordination interactions and electrostatic self-assembly. Their experimental results demonstrated that these nanoparticles efficiently disassembled intracellular Aβ oligomers and fibrils, increasing the survival rate of Aβ-induced PC12 cells from 60% to 85%. Furthermore, experimental results demonstrated that FTFQ NPs were more effective than donepezil in alleviating symptoms in Alzheimer’s disease (AD) zebrafish. Following injection of DCFH/ThT fluorescent probes, clearance of ROS and Aβ in the brains of AD zebrafish was observed, indicating that FTFQ NPs eliminated more ROS and Aβ.

Nicholas et al. [98] designed gold nanoparticles coated with an EGCG and Zn (II) coordination network (MPN@AuNP) to efficiently inhibit Aβ aggregation and toxicity. At physiological concentrations, Zn^2+^ not only exerts neuroprotective effects but also competitively binds to histidine residues of Aβ, thereby interfering with Cu^2+^/Fe^3+^-induced Aβ aggregation and reactive oxygen species (ROS) generation. Furthermore, using the hCMEC/D3 endothelial cell model, the researchers confirmed that these nanoparticles can traverse the blood–brain barrier (BBB) via the paracellular pathway, providing a novel metal-chelating delivery strategy for the treatment of Alzheimer’s disease (AD).

### 3.4. Synergistic Strategies Against Drug Resistance and Immune Evasion in the Glioblastoma Microenvironment

#### 3.4.1. Mechanisms of Glioma Drug Resistance: Apoptosis Resistance and DNA Damage Repair

The intractability of glioblastoma (GBM) primarily stems from its highly invasive nature, intrinsic drug resistance, and robust immunosuppressive microenvironment. The therapeutic efficacy of the conventional chemotherapeutic agent temozolomide (TMZ) is severely compromised by multiple resistance mechanisms, including the following: (1) DNA repair—TMZ induces O^6^-methylguanine DNA damage; however, GBM cells can directly counteract this effect by upregulating O^6^-methylguanine-DNA methyltransferase (MGMT) to repair the lesions, rendering the drug ineffective [99]. (2) Efflux pumps [100]—Drug-resistant cells overexpress ATP-binding cassette (ABC) transporters, such as P-glycoprotein (P-gp), which actively extrude TMZ from the cells, thereby conferring drug resistance. (3) Apoptosis resistance and ferroptosis evasion—GBM cells acquire resistance to apoptosis and ferroptosis by upregulating anti-apoptotic proteins (e.g., Bcl-2) and enhancing the GPX4/GSH antioxidant axis [101]. (4) Physical barriers—The blood–brain barrier (BBB) restricts drug entry into the brain, while the dense tumor matrix and abnormal vasculature further impede drug penetration within the tumor mass [102].

#### 3.4.2. MPN-Mediated Metabolic Intervention, Ferroptosis Induction, and cGAS-STING Pathway Activation

To counteract the aforementioned multifaceted drug resistance and immune evasion mechanisms, the primary strategies employed by MPNs include the following: (1) Synergistic metabolic intervention and ferroptosis induction to overcome drug resistance—Metal ions (e.g., Fe^3+^) are utilized to trigger ferroptosis, thereby eliminating drug-resistant cells that are insensitive to apoptosis [103]. Concurrently, this approach is combined with the inhibition of metabolic pathways (such as pyrimidine synthesis) to deplete DNA repair proteins (MGMT) and block drug efflux, fundamentally reversing chemoresistance. (2) Induction of immunogenic cell death (ICD)—By integrating sonodynamic therapy (SDT) [104] and chemodynamic therapy (CDT) [15], MPNs generate substantial amounts of reactive oxygen species (ROS) to induce ICD in tumor cells. (3) Activation of the cGAS-STING innate immune pathway [105]—Metal ions such as Mn^2+^ act synergistically with cytosolic DNA fragments to potently activate the cGAS-STING pathway. This promotes the secretion of type I interferons, thereby converting tumor cell death into robust endogenous immune-activating signals and eliciting durable antitumor T-cell responses.

For instance, the study by Na Yin et al. [106] systematically reversed TMZ resistance through a triple synergistic mechanism. This platform inhibited DHODH to deplete MGMT, thereby blocking DNA damage repair. Concurrently, Fe^3+^ was utilized to disrupt the DHODH/GPX4 defense system, triggering robust lipid peroxidation and ferroptosis. Additionally, the cMBP peptide was employed to suppress the MET pathway, which downregulated drug efflux pumps and consequently increased the intracellular concentration of TMZ. Furthermore, surface modification with the T10 peptide facilitated blood–brain barrier (BBB) crossing and glioma targeting by hijacking the transferrin transport pathway.

Zhiqiang Wang et al. [61] constructed a biomimetic nanomicelle featuring an elastin-like polypeptide (ELP) micellar core loaded with the sonosensitizer Ce6 (for SDT), a Mn^2+^-TA coordination network intermediate layer (for CDT and MRI), and an outermost macrophage membrane coating (reported to promote BBB crossing and glioma targeting). Upon ultrasound irradiation, the combined SDT/CDT-induced oxidative stress triggered immunogenic cell death (ICD) and caused DNA damage. Simultaneously, the Mn^2+^ ions activated the cGAS-STING pathway, promoting type I interferon secretion and dendritic cell maturation. This nanomicelle successfully elicited a robust antitumor immune response in an orthotopic GBM mouse model, significantly prolonging the median survival time and establishing long-term immunological memory.

Yulin Zhang et al. [107] developed a multifunctional metal–phenolic network nanoparticle platform, termed cRGD/Pt+DOX@GFNPs, for the multi-targeted combination therapy and magnetic resonance (MR) imaging-guided treatment of glioblastoma. This platform synergistically disrupted the redox homeostasis of tumor cells through multiple mechanisms, including the Fenton reaction (mediated by Fe^2+^), chemotherapy (DOX and Pt), and near-infrared (NIR) photothermal therapy. These concerted actions simultaneously induced apoptosis and ferroptosis, demonstrating the potent efficacy of combination therapy.

The preceding sections have reviewed various MPN platforms in different brain disease models. To facilitate a cross-study comparison of the representative MPN described above and to highlight differences in the level of evidence among these studies, we summarize the key attributes of these MPN formulations in Table 3, with a focus on their MPN composition, disease models, blood–brain barrier (BBB) crossing evidence (both in vivo and in vitro), therapeutic mechanisms, primary outcomes, biosafety, and limitations.

### 3.5. Prospective Design of MPN Targeting NETs for the Treatment of Brain Diseases

The content discussed in this section represents future research directions proposed by the authors, rather than established therapeutic strategies that have been experimentally validated. Therefore, this section aims to construct a prospective conceptual framework based on existing NET research and the technological advantages of MPNs, providing a theoretical foundation and reference for experimental design for subsequent studies.

#### 3.5.1. Pathological Evidence of NETs in Brain Diseases

**Figure 7:**
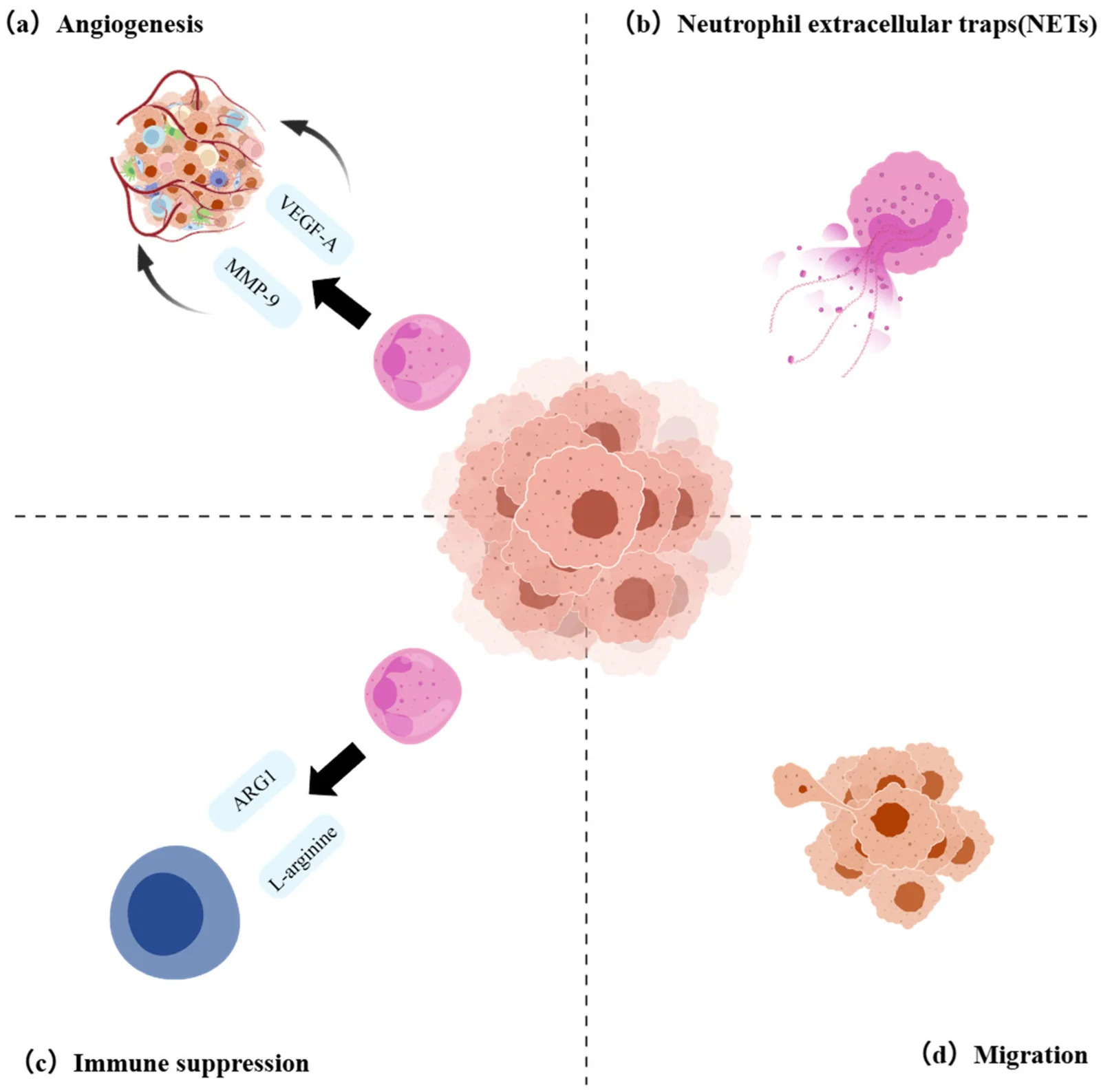
A schematic overview of the pro-tumor activities of tumor-associated neutrophils (TANs) in the glioblastoma microenvironment. (**a**) Angiogenesis: N2-like TANs drive aberrant neovascularization by releasing vascular endothelial growth factor (VEGF-A) and matrix metalloproteinase-9 (MMP-9), promoting endothelial sprouting and extracellular matrix remodeling. (**b**) Immunosuppression: TANs suppress adaptive immunity through arginase-1 (ARG1)-mediated depletion of L-arginine, leading to impaired T-cell proliferation and effector function. (**c**) NETosis: The formation of neutrophil extracellular traps (NETs), composed of chromatin, proteases, and reactive oxygen species (ROS), promotes tumor invasion, increased vascular permeability, blood–brain barrier disruption, and immune evasion. (**d**) Migration: Chemokine-driven neutrophil trafficking and retention within hypoxic and perivascular niches support tumor infiltration, spatial organization, and sustained pro-tumor signaling.

Neutrophil extracellular traps (NETs) are extracellular web-like structures released by neutrophils in response to stimuli within the tumor microenvironment [108]. They consist of a decondensed chromatin backbone embedded with granular proteins such as myeloperoxidase (MPO), neutrophil elastase (NE), and citrullinated histone H3 (CitH3). Within the complex microenvironment of glioblastoma (GBM), tumor cells can induce neutrophils to form NETs [109,110]. These structures not only constitute a physical barrier that impedes drug penetration but also play an important role in facilitating tumor immune evasion and proliferation (Figure 7).

**Figure 8:**
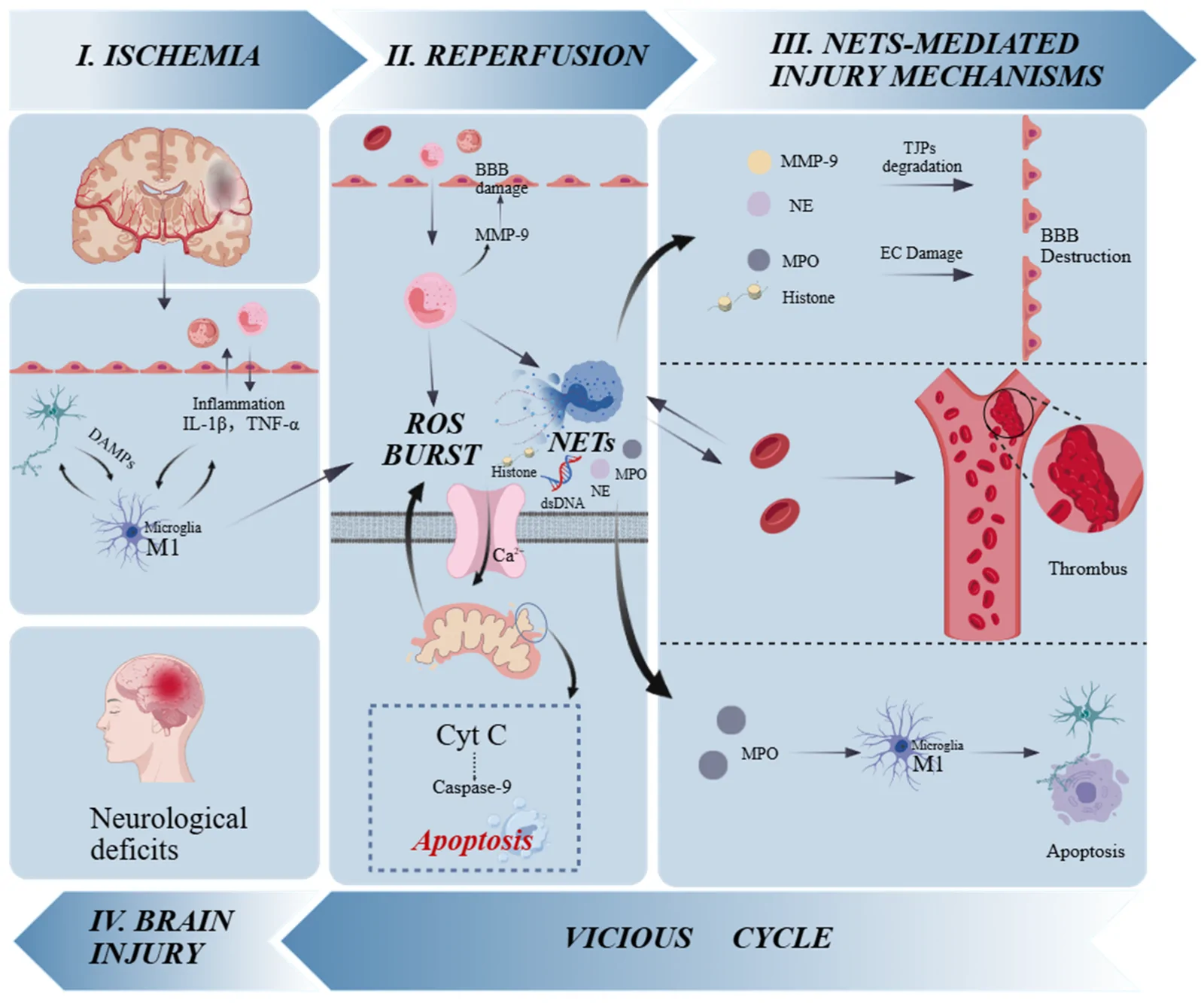
NETs exacerbate neuroinflammation, thrombosis, and blood–brain barrier disruption through ROS-dependent NETosis, thereby aggravating brain injury.

NETs also play a critical role in ischemic stroke: activated neutrophils release NETs through ROS-dependent NETosis, exacerbating neuroinflammation, thrombosis, and blood–brain barrier disruption, thereby aggravating brain injury [111]. Therefore, inhibiting NET formation has become an important therapeutic strategy and can be achieved by interfering with ROS generation, scavenging free radicals, or inhibiting key enzymes such as peptidylarginine deiminase 4 (PAD4) (Figure 8).

In patients with Alzheimer’s disease (AD), neutrophil accumulation in cerebral vessels and parenchyma is closely associated with Aβ plaques and neurofibrillary tangles (NFTs); Aβ can induce NETosis; NETs and their released pro-inflammatory factors exacerbate Aβ deposition, NFT formation, chronic inflammation, and cognitive impairment [112].

#### 3.5.2. Potential Strategies of MPNs Targeting NETs

Given the critical role of NETs in these three diseases and the inherent bioactivity-related advantages of MPNs, leveraging MPNs to inhibit NETs represents a highly promising therapeutic direction for the treatment of brain diseases. Although no direct studies on MPN-based targeting of NETs for brain diseases have been reported to date, potential design strategies can be inferred based on existing research, as outlined below (Table 4).

## 4. Future Perspectives and Translational Challenges

Metal–phenolic networks (MPNs) have broad therapeutic potential and have demonstrated applicability across various disease models. In the field of oncology, researchers have constructed various multifunctional nanoplatforms that integrate chemodynamic therapy (CDT), photothermal therapy (PTT), sonodynamic therapy (SDT), immunotherapy, and ferroptosis induction for the treatment of glioblastoma [106] and breast cancer [116], achieving significant tumor suppression in animal models. In the realm of tissue regeneration and repair [117], MPN coatings have been applied to orthopedic implants to promote osteogenesis and angiogenesis, and they have also been developed as chronic wound dressings, leveraging their antibacterial, anti-inflammatory, and vascular normalization properties to accelerate healing. Furthermore, in the treatment of central nervous system (CNS) disorders [118] such as Alzheimer’s disease (AD) and ischemic stroke, MPNs have shown considerable promise in scavenging reactive oxygen species (ROS), modulating neuroinflammation, and promoting neural repair.

MPNs can be flexibly engineered into various forms, including nanoparticles, coatings, hydrogels, and capsules, to accommodate diverse clinical needs [118]. For instance, they can be utilized as nanoparticles for systemic delivery or as coatings to endow medical device surfaces with anti-infective and pro-tissue-integration properties [117,119]. More importantly, MPNs can readily integrate imaging modalities (e.g., magnetic resonance imaging, fluorescence imaging, and photoacoustic imaging) with multiple therapeutic mechanisms, enabling the use of theranostics—a powerful tool for advancing precision medicine [116].

Furthermore, a growing number of studies have begun to focus on the key elements essential for clinical translation. These studies have investigated the in vivo biodistribution, metabolic pathways, and clearance routes of MPNs and evaluated their long-term biosafety [3]. Surface modifications, such as PEGylation and targeted peptide conjugation, are employed to improve pharmacokinetic profiles and targeting capabilities while reducing off-target toxicity [119]. Notably, although several MPN building blocks, such as tannic acid and gallic acid, are already FDA-approved food additives, this GRAS (Generally Recognized as Safe) status is established on the basis of oral dietary exposure and cannot be extrapolated to intravenously administered metal-coordinated nanomedicines, whose dose, route of exposure, bioavailability, specific biodistribution, and long-term accumulation toxicity differ fundamentally from those of ingested food ingredients [120]. Therefore, any MPN therapeutic must be developed as a new drug entity under a complete IND (Investigational New Drug)-enabling program, including GMP-compliant manufacturing and Chemistry, Manufacturing, and Controls (CMC) documentation [121].

A pivotal yet frequently underappreciated determinant of both efficacy and safety is the relationship between coordination stability, metal release kinetics, and therapeutic activity. Upon intravenous administration, MPNs traverse two chemically opposing environments. In serum (pH 7.4), the coordination network is challenged by two competing ligands—albumin and transferrin—which can exchange with phenolate donors and trigger premature disassembly [122,123]. Premature metal release is doubly detrimental: it causes off-target exposure of free metal ions in the systemic circulation and depletes the formulation before it reaches the brain lesion. Conversely, in the endo-/lysosomal compartment of target cells (pH 4.5–6.0), the combined action of phosphate, citrate, and glutathione labilizes metal–phenolate bonds and accelerates network disassembly; for most CNS-oriented MPNs, this acidic compartment is precisely the intended site of action, where the released ligands and ions execute Fenton-based CDT or ferroptosis induction [124,125]. Therefore, MPNs that are too labile disassemble in serum and forfeit their targeting advantage, whereas overly inert networks fail to release their payload and may persist as non-degradable deposits. The key to achieving therapeutic efficacy with MPNs lies in their mechanism-dependent design. CDT/ferroptosis-oriented platforms require lysosome-labile coordination, so that catalytically silent Fe^3+^ is released and reduced in situ to Fe^2+^; antioxidant- and chelation-oriented platforms demand intermediate stability that preserves the polyphenol–metal complex long enough during circulation and tissue distribution to scavenge reactive oxygen species (ROS) and sequester labile metals [126]; and structural coating or imaging platforms favor higher kinetic stability. Therefore, quantitative release profiling under simulated serum and lysosomal conditions should become a standard element of MPN preclinical evaluation.

Despite the immense preclinical potential demonstrated by MPNs, their clinical translation pathway remains fraught with formidable challenges. Based on data retrieved from major clinical trial registry platforms, numerous polyphenol- or metal-based agents have been assessed in clinical studies (data sourced from ClinicalTrials.gov and https://trialsearch.who.int/ (accessed on 23 July 2026)). For instance, copper has been investigated for the treatment of patients with mild Alzheimer’s disease or brain tumors (NCT00608946; NCT04488783). Additionally, zinc has been employed in combination regimens for glioma patients (NCT01777919; NCT02715609). Furthermore, clinical trials have been conducted to evaluate the antioxidant and anti-inflammatory activities of polyphenols in a range of diseases (NCT07284693; PACTR202509489915993; TCTR20180419005; IRCT2016101517756N9). However, no drug or delivery system explicitly designated as a metal–phenolic network has yet advanced to human clinical trials. Consequently, while MPN research has yielded substantial laboratory advances, translating these findings into practical clinical applications necessitates overcoming a series of significant hurdles (Table 5).

Among these challenges, manufacturing and quality control deserve particular attention, because clinical application will ultimately rest on demonstrable batch-to-batch consistency of the critical quality attributes (CQAs) of the final product. Under a quality-by-design (QbD) framework (ICH Q8–Q11), the CQA panel for an injectable MPN should include: (i) the metal-to-ligand coordination stoichiometry, the key determinant of network density and release behavior, quantified by inductively coupled plasma optical emission spectrometry (ICP-OES) or inductively coupled plasma mass spectrometry (ICP-MS) combined with elemental or thermogravimetric analysis; (ii) the oxidation state of the metal node, which governs Fenton or Fenton-like activity and must be verified by X-ray photoelectron spectroscopy; (iii) hydrodynamic diameter and polydispersity index, monitored by dynamic light scattering with orthogonal confirmation by transmission electron microscopy; (iv) zeta potential, which indicates colloidal stability, protein corona formation, and cellular uptake; (v) residual free metal content, which must be minimized through validated purification processes (dialysis, ultrafiltration, or size-exclusion chromatography), because labile metal is a direct contributor to off-target toxicity; (vi) drug loading and encapsulation efficiency for cargo-bearing formulations; (vii) in vitro release profiles under both serum-mimicking (pH 7.4) and lysosomal-mimicking (pH 4.5–5.5) conditions, as discussed above; and (viii) product safety attributes, including endotoxin (LAL test, USP <85>), sterility (USP <71>), and bioburden. Because the protein corona acquired in serum constitutes the actual biological identity of the injected particle, standardized characterization of corona composition by SDS-PAGE and LC-MS/MS is increasingly regarded as an integral part of release testing. At the manufacturing scale, MPN self-assembly is acutely sensitive to pH, ionic strength, mixing rate, and reactant concentration, so conventional batch mixing scales poorly; microfluidic reactors coupled with in-line process analytical technology (real-time dynamic light scattering, UV-vis, and pH monitoring) provide tighter control over nucleation and growth and markedly improve batch-to-batch reproducibility from laboratory to kilogram-scale GMP production. Finally, aqueous MPN dispersions are susceptible to oxidative and hydrolytic aging; lyophilized formulations with appropriate cryoprotectants and lyoprotectants, validated by real-time and accelerated stability studies under ICH Q1A (R2), will be required to define shelf life, storage conditions, and reconstitution performance.

## 5. Conclusions

In this review, we provide a comprehensive overview and in-depth analysis of the current research status and future directions of metal–phenolic networks (MPNs) in the treatment of brain diseases. As a highly promising class of nanomaterials, MPNs have demonstrated immense potential in preclinical studies for addressing challenges related to complex diseases. However, no MPNs have yet entered clinical investigation. Therefore, bridging the gap from laboratory research to clinical application necessitates continued in-depth investigation in several key areas, including simplifying and standardizing the fabrication processes, thoroughly elucidating their in vivo metabolic profiles and long-term safety profiles, developing precise targeted delivery strategies, and exploring viable clinical translation and regulatory pathways.

## Figures and Tables

**Table 1:** Function-oriented selection of metal ion nodes in metal–phenolic networks (MPNs).

Metal Category	Representative Ions	Key Functions & Mechanisms	Direct Evidence for MPNs	Refs
Transition metals	Fe^2+/3+^, Cu^+/2+^, Mn^2+^	CDT: Fe and Cu catalyze Fenton/Fenton-like reactions, converting H_2_O_2_ to ·OH in tumor/inflammatory microenvironments. ROS scavenging & MRI: Mn^2+^ cycles between oxidation states (Mn^2+/3+/4+^) to scavenge ROS and serves as a T^1^ MRI contrast agent for theranostics.	CDT: Fe-TA MPN exhibits pH-dependent ·OH generation in vitro and demonstrates tumor-suppressive effects in GBM models. GA-Cu MPN exhibits effective mucus penetration and eliminates drug-resistant bacteria through a cuproptosis-like mechanism. ROS scavenging & MRI: Sr^2+^/Mn^2+^@Rh MPN exhibits ROS scavenging and M2-type microglial polarization in ischemic stroke models; Mn-TA MPN enables MRI imaging in bladder cancer.	[13,14,15,16,17,18]
Biologically essential metals	Zn^2+^, Mg^2+^, Sr^2+^	Neuroprotection: Zn^2+^ inhibits NMDA receptor-mediated excitotoxicity; Mg^2+^ modulates NMDA receptors, Ca^2+^ balance, and mitochondrial protection to alleviate secondary nerve injury. Angiogenesis: Sr^2+^ promotes endothelial proliferation and vascular normalization in ischemic regions.	Neuroprotection: There is no direct evidence for Zn^2+^ and Mg^2+^ crossing the BBB as intact MPN. Angiogenesis: Sr^2+^/Mn^2+^@Rh MPN exhibits vascular normalization	[19,20,21]
Rare-earth metals	Gd^3+^, Yb^3+^	MRI tracking: Gd^3+^ enables real-time, non-invasive T^1^ MRI monitoring of nanoparticle biodistribution. Multimodal imaging: Yb^3+^ provides NIR fluorescence and CT imaging capabilities for imaging-guided therapy.	MRI tracking: Gd^3+^ has been incorporated into the MPN framework for T_1_ MRI imaging; BaGdF_5_@TA-Eu MPN enables multimodal imaging. Multimodal imaging: Yb^3+^-coordinated MPN exhibits near-infrared emission and CT imaging capabilities.	[22,23,24,25,26]

CDT, chemodynamic therapy; CT, computed tomography; H_2_O_2_, hydrogen peroxide; MRI, magnetic resonance imaging; NIR, near-infrared; NMDA, *N*-methyl-*D*-aspartate; ·OH, hydroxyl radical; ROS, reactive oxygen species.

**Table 2:** BBB-crossing strategies.

Evidence Level	Meaning	Corresponding Strategies
Direct crossing	Intact particles cross the BBB and enter the brain parenchyma	Ligand–receptor-mediated transcytosis
Increased accumulation	Demonstrates increased brain accumulation/enrichment, which may originate from leakage at disrupted barriers rather than active crossing	Macrophage membrane biomimetics, ultrasmall particle size
Design hypothesis	Based on mechanistic inference, lacking direct experimental support	Extrapolation from EPR effect and polyphenol molecular permeability to MPN crossing

**Table 3:** MPN platforms.

MPN	MPN Composition	Disease Model	BBB Crossing Evidence (In Vitro)	BBB Crossing Evidence (In Vivo)	Therapeutic Mechanism	Results	Biosafety	Limitations
Sr_2_/Mn_2_@Rh	Metals: Mn^2+^ + Sr^2+^; Polyphenol: Rutin; PVP as surfactant	Cellular OGD/R: SH-SY5Y, BV2, bEnd.3; Animal: tMCAO SD rats	bEnd.3/SH-SY5Y co-culture system demonstrated BBB penetration and neuronal internalization	No in vivo biodistribution, imaging, or brain quantification evidence; Brain accumulation inferred from nanosize effect	Acid-triggered MPN disassembly: Mn^2+^/Mn^3+^ redox cycling for ROS scavenging; Rh drives microglial M1 to M2 polarization; Sr^2+^ promotes endothelial migration and angiogenesis; weak-base ions neutralize acidic microenvironment; CASP-3/GFAP/IBA-1/MPO/LC3 downregulated	DPPH and ABTS scavenging rates 83.4% and 94.6%; Rh release 71.2% at pH 5.5 (9 h); reduced neuronal death under OGD/R, iNOS downregulated with CD206 upregulation, bEnd.3 tube formation restored; in vivo: reduced brain tissue damage and neurological deficits; decreased CASP-3, GFAP, IBA-1, MPO, LC3 levels	Cell viability >80% at 200 μg/mL; no hemolysis; normal organ H&E after 3 d treatment; normal blood routine, biochemistry, H&E in healthy rats at 14 d	No direct in vivo biodistribution evidence for BBB penetration; BBB disruption in tMCAO model may facilitate passive accumulation
GQDs@MPN	Metal: Co^2+^; Polyphenol: EGCG; Core–shell structure coated on GQDs	AD model; Cells: PC12, BV2, HUVEC, bEnd.3; Animals: APP/PS1 transgenic mice and healthy KM mice	GQDs@MPN crossed BBB via bEnd.3/PC12 co-culture, proposed to be mediated by GLUT1 glucose transporter; HUVEC uptake observed	Tail-vein injection in healthy KM mice; ex vivo brain hippocampal fluorescence increased from 1–12 h, peaking at 6 h, indicating crossing of non-disrupted vasculature	Inhibits Aβ_42_ aggregation; effective ROS scavenging; blocks Aβ-membrane binding; microglial M1→M2 polarization; Nrf2 upregulation, NF-κB pathway inhibition; enhances Aβ phagocytosis	β-sheet content decreased from 50.4% to 28.7%; Aβ_42_ binding Kd 39.63; PC-12 viability increased to 95.2%; ·O_2_^-^ scavenging 90.1%; BV2 apoptosis decreased from 47.7% to 20.9%; improved nesting behavior in APP/PS1 mice; neuronal protection (Nissl staining showed neuronal density recovery); plaque area reduced to 3.4%, Iba-1/TNF-α decreased, Nrf2 upregulated	Cell viability >99.5% at 5–400 μg/mL; low hemolysis rate; no organ damage on H&E staining	GLUT1 mechanism for BBB crossing is hypothetical based on surface glucose groups, lacking direct inhibitor validation; no long-term or cobalt-specific safety assessment; authors call for dose optimization/prolonged treatment
TQCN	Metal: Fe^3+^; Polyphenol: Quercetin; TPP mitochondria targeting	HT22 ferroptosis model; APP/PS1 transgenic mice	Not reported	Intranasal administration bypasses BBB; Cy5.5 brain accumulation peaked at 2 h; colocalization with neurons and mitochondria in hippocampus/cortex	TPP targets neuronal mitochondria; QC chelates excess iron and in situ assembles into nanocomplexes, enhancing radical scavenging; iron chelation inhibits Fenton reaction; activates Nrf2/GPX4 pathway to inhibit ferroptosis; upregulates FPN1/FTH1 to restore iron homeostasis	HT22 cell viability restored; apoptosis decreased to 15.8%; in vivo reduced brain iron deposition; cognitive function restored (OFT, NOR, Y-maze, MWM)	In vitro: no significant neurotoxicity; In vivo: serum biochemistry, blood routine, H&E staining showed no obvious abnormalities; QC is natural molecule, PEG is FDA-approved excipient, TPP is non-toxic	No direct BBB crossing evidence (intranasal bypasses BBB); metabolic products and long-term safety of TQCN require further investigation
FTFQ NPs	Polyphenol: Quercetin; Metal: Fe^2+^	Aβ-induced PC12 cells; AlCl_3_-induced AD zebrafish	Indirect: BBB penetration inferred from literature-reported size (<250 nm)	Not reported	Inhibits BACE1 activity; reduces Aβ aggregation; induces Aβ fibril disaggregation; scavenges ROS; Fe^2+^ stably chelated to avoid Fenton toxicity	Cell survival increased to 85% under Aβ induction; fibrillation inhibition 80.5%, disaggregation 82.3%, BACE1 inhibition 63.6%; zebrafish mobility restored to 90%	No zebrafish malformation at 0–100 μg/mL	No mammalian models; BBB not directly demonstrated; Fenton reaction risk from Fe^2+^ not fully evaluated
MPN@AuNP	Polyphenol: EGCG; Metal: Zn^2+^	hCMEC/D3 BBB model; Aβ_1-42_-induced SH-SY5Y neurotoxicity	hCMEC/D3 Transwell; crossed BBB via paracellular route through hCMEC/D3 monolayer (NanoEL, VE-cadherin gap formation)	Not reported; no animal experiments	Aβ sequestered into MPN mesoporous channels via hydrogen bonding and aromatic stacking; inhibits Aβ fibrillation (ThT, FTIR, DMD simulation)	Inhibited Aβ fibrillation (ThT fluorescence dose-dependent decrease); reduced Aβ-induced ROS and cytotoxicity (SH-SY5Y viability increased to 84%); NanoEL without cytotoxicity	EGCG@AuNP and MPN@AuNP showed no cytotoxicity at 12.5–100 μM EGCG; Zn(II) release did not cause significant ROS; NanoEL without cell death; dual anti/pro-oxidant effects of EGCG noted	Long-term in vivo accumulation risk of AuNP not assessed; BBB crossing verified only in vitro; relationship between Zn(II) concentration and AD brain levels not elucidated
RPDGs	Polyphenol: Gallic acid; Metal: Fe^2+^	U87MG cells; orthotopic GBM nude mouse model; C57BL/6 for biosafety	Not reported	cRGD recognizes αvβ_3_ integrin, mediating BBB crossing and tumor targeting (confirmed by in vivo fluorescence tracing)	CDT: Fe^2+^ release sustains Fenton reaction; Pt(IV) consumes GSH increasing ROS, synergizing with DOX; NIR photothermal therapy; cRGD targets αvβ_3_; Fe^2+^ for T_2_ MRI	Significantly inhibited orthotopic GBM growth; median survival extended from 17.2 to >52.8 days; MRI tracking (r_2_ = 81.0 mM^−1^s^−1^); photothermal effect (808 nm)	In vivo H&E showed no major organ damage; blood routine and biochemistry normal; RPDGs showed good biosafety	Multiple therapeutic mechanisms increase toxicity risk; NIR irradiation increases intracranial temperature (35.5 °C) potentially affecting normal brain function; long-term Fe/Pt fate unresolved
Ce6-ELP@TM/MM	Metal: Mn^2+^; Polyphenol: Tannic acid	C6 rat glioma cells, bEnd.3, DCs; orthotopic C6 glioma mouse model; healthy mice for biosafety	bEnd.3 Transwell: Ce6-ELP@TM/MM+US > +MM > TM > ELP penetration; attributed to MM-VCAM-1 interaction and US cavitation	Indirect: macrophage membrane coating reported to enhance brain accumulation and tumor homing; TA hydroxyl groups in TM may assist BBB penetration	ICD: Mn^2+^ Fenton-like CDT, Ce6 generates ROS under US (SDT) causing mitochondrial damage, inducing DNA damage; Mn^2+^ synergistically activates cGAS-STING → IFN-β, DC maturation, CD8^+^ T-cell infiltration	Significantly inhibited orthotopic GBM growth (confirmed by MR imaging); DC maturation increased 2.7-fold; tumor-infiltrating CD3^+^CD4^+^ and CD3^+^CD8^+^ T cells significantly increased; Tregs decreased; immune memory established	In vivo H&E showed no major organ damage; blood routine and biochemistry normal; US irradiation (2 W/cm^2^, 2 min) increased brain temperature ~3 °C without significant damage	No long-term Mn toxicity; US irradiation conditions (2 W/cm^2^, 2 min) may produce thermal effects on normal brain tissue
TBFP-MT	Metal: Fe^3+^; Polyphenol: PEG-polyphenol	TMZ-resistant GBM; GL261/GL261R cells; orthotopic GL261R glioma in C57BL/6 mice	In vitro BBB model showed TBFP-MT penetration ~3-fold higher than non-T10-modified group	Not directly reported; T10 peptide binds serum transferrin (Tf) to form protein corona, crossing BBB via TfR, but no in vivo biodistribution or ICP-MS brain iron quantification	DHODH inhibition blocks de novo pyrimidine synthesis, specifically downregulating MGMT expression, blocking DNA damage repair; Fe^3+^ triggers ferroptosis, disrupting DHODH/GPX4 dual defense axis; cMBP inhibits MET pathway, downregulating efflux pumps and increasing TMZ intracellular accumulation; T10/TfR mediates BBB crossing and GBM targeting	Significantly inhibited orthotopic TMZ-resistant GBM growth (T_2_-MRI dynamic monitoring over 15 days); tumor H&E showed obvious necrosis and karyolysis; median survival significantly prolonged; cell viability <20% (at 50 μg/mL TMZ equivalent)	PEG-polyphenol no cytotoxicity at 400 μg/mL; H&E showed no major organ damage (heart, liver, spleen, lung, kidney); serum biochemistry (AST, ALT, CRE) normal	T10 peptide mechanism relying on Tf corona is novel, but TfR is also expressed in normal brain tissue, posing potential off-target risk; long-term metabolic effects of DHODH inhibitors not fully evaluated; persistence of MGMT downregulation in TMZ-resistant model not tracked

**Table 4:** Potential MPN-based design strategies for targeting neutrophil extracellular traps (NETs).

Design Strategy	Key Approach	Mechanism & Rationale	Refs
Inhibition of NET formation	PAD4 inhibitor-loaded MPN	Targeted delivery: MPN loaded with PAD4 inhibitors block histone citrullination and chromatin decondensation, suppressing NET generation. pH-responsive release: MPNs ensure selective drug release within the acidic tumor microenvironment, minimizing systemic immune interference. Stroke: PAD4 inhibition reduces NET formation, improves microvascular perfusion, and decreases infarct volume in experimental stroke models. AD: PAD4 inhibition reduces Aβ deposition and cognitive decline; genetic or pharmacological inhibition of PAD4 alleviates AD pathology.	[113,114]
Degradation of pre-existing NETs	DNase I-encapsulated MPN	GBM: Encapsulated DNase I specifically degrades the DNA backbone of NETs at tumor sites. Stroke: Degrades the NETs scaffold in stroke thrombi and perivascular NETs, alleviating the no-reflow phenomenon and BBB disruption. AD: Degrades perivascular NETs in cerebral vessels, reducing Aβ-induced vascular damage and neuroinflammation.	[98,115]
Intrinsic NET-inhibitory activity of polyphenolic ligands	EGCG, resveratrol, curcumin (as MPN ligands)	ROS scavenging: EGCG directly blocks NET release by scavenging ROS and inhibiting MPO activity. Pathway suppression: Resveratrol reduces NET formation via the ROS/PAD4 pathway; curcumin blocks NETosis by downregulating PAD4 expression. Intrinsic advantage: As EGCG and TA are common MPN ligands, polyphenols released upon MPN dissociation may exert analogous NET-inhibitory effects at lesion sites.	[99,100,101,102,103]
Synergistic therapeutic enhancement	NET degradation + chemo/immunotherapy	GBM: NET degradation enhances penetration of chemotherapeutic agents (e.g., TMZ) into tumor parenchyma. Relieving immunosuppression amplifies ICD-induced immune responses; exposed tumor antigens synergize with cGAS-STING activation to potentiate adaptive antitumor immunity. Stroke: NET degradation combined with anti-inflammatory therapy synergistically alleviates reperfusion injury and neuroinflammation. AD: NET degradation combined with Aβ immunotherapy synergistically clears Aβ and alleviates chronic inflammation.	[104]

cGAS-STING, cyclic GMP-AMP synthase-stimulator of interferon genes; EGCG, epigallocatechin gallate; GBM, glioblastoma; ICD, immunogenic cell death; MPN, metal–phenolic network; MPO, myeloperoxidase; NETs, neutrophil extracellular traps; PAD4, peptidylarginine deiminase 4; ROS, reactive oxygen species; TA, tannic acid; TMZ, temozolomide.

**Table 5:** Key translational challenges for clinical application of metal–phenolic networks (MPNs).

Translational Challenge	Core Issue	Key Details & Rationale
Long-term safety & biodegradability	Chronic metal accumulation; immune activation	Metabolic fate: The long-term in vivo metabolic fate of MPN assemblies remains poorly elucidated. Metal accumulation: Although building blocks (e.g., tannic acid, EGCG, Fe^3+^, Mn^2+^) are generally low in toxicity, metal ions may chronically accumulate in the liver and kidneys, potentially triggering adverse effects. Immune activation: At the nanoscale, MPNs may activate the complement system or induce immune cell phagocytosis, resulting in unpredictable immune responses. Required action: Systematic long-term toxicological and immunotoxicological assessments are essential.
Preparation stability & quality control	Batch inconsistency; protein corona formation	Assembly sensitivity: MPN self-assembly is highly sensitive to pH, ionic strength, and mixing speed, making stringent batch-to-batch consistency (particle size, surface potential, drug loading) difficult to achieve during scale-up. Protein corona: Upon entering systemic circulation, MPN surfaces may rapidly adsorb proteins, forming a *protein corona* [108], which can alter targeting specificity and pharmacological activity, complicating precise evaluation of therapeutic efficacy.
Large-scale manufacturing	Lab-to-GMP scale-up; purification complexity	Scale-up gap: Transitioning from milligram-scale laboratory preparation to kilogram-scale GMP-based production remains a substantial engineering challenge. Process control: Advanced microfluidic or continuous-flow synthesis technologies are required to precisely control the assembly process. Purification: Mild purification techniques that do not compromise fragile coordination structures must be established to achieve high-purity, sterile, and cost-effective clinical-grade production.

EGCG, epigallocatechin gallate; Fe^3+^, ferric ion; GMP, Good Manufacturing Practice; Mn^2+^, manganese ion; MPN, metal–phenolic network.

## Data Availability

No new data were created or analyzed in this study. Data sharing is not applicable to this article.
